# Development and validation of a questionnaire for table tennis teaching in physical education

**DOI:** 10.3389/fpsyg.2025.1550061

**Published:** 2025-01-24

**Authors:** Miguel Ángel Ortega-Zayas, Antonio José Cardona-Linares, Alberto Quílez, Alejandro García-Giménez, Miguel Lecina, Francisco Pradas

**Affiliations:** ^1^ENFYRED Research Group, Faculty of Health and Sports Sciences, University of Zaragoza, Huesca, Spain; ^2^Faculty of Human Sciences and Education, University of Zaragoza, Zaragoza, Spain; ^3^Faculty of Health Sciences, ENFYRED Research Group, University of Zaragoza, Zaragoza, Spain

**Keywords:** racket sports, questionnaire, physical education, teacher skills, educational content

## Abstract

This study aimed to validate the “Racquet Sports Attitude Scale (RSAS)” questionnaire intended for the educational field. The RSAS is a Spanish instrument developed using the Delphi method and consists of 42 questions divided into five subsections benefits of table tennis (TT), facilitators for the application of TT, barriers to applying tennis table in physical education (PE), difficulties in applying the tennis table as a PE content and positive attitude toward racket sports), designed to evaluate the use and impact of the practice of the racket sports, in particular TT, among PE teachers. The semantic validation process involved a panel of experts (*n* = 16), followed by an evaluation carried out by a group of teachers from various secondary schools (*n* = 20). Three studies were conducted, the first of which was descriptive, comparative and correlational, to establish the data’s sociodemographic characteristics. The second was an exploratory factor analysis to confirm the number of dimensions of the questionnaire and its reliability and validity. The last study was a confirmatory factor analysis (to establish the final number of items and their structure. The ordinal alpha values ranged from 0.65 to 0.72, obtaining an average value of 0.70, indicating coherence between the measurement of the items. Chi-square values (X^2^ = 197.383; *p* < 0.001; CFI = 0.934; TLI = 0.923 and RMSEA = 0.030), showed that the original 5-factor model and 21 final items prevailed over the one-factor model, obtaining values >0.90. On the other hand, the validation results indicated that the factor structure of the RSAS scale was able to explain 46.72% of the variance (F1 = 13.93%; F2 = 10.97%; F3 = 8.57%; F4 = 6.85%; F5 = 6.38). It can be concluded that the structure of the RSAS is fully confirmed by the statistical analyses derived from the different models of structured equations and confirmatory factor analysis, suggesting that the validated model optimally addresses the data provided and contributes to the validation of the questionnaire under study.

## 1 Introduction

Racket sports (RS) are regulated and institutionalized disciplines that appeared throughout the twentieth century to respond to a growing social demand for alternative sports that differ from traditionally practiced sports. This family of sports, among which some as popular and consolidated as badminton, tennis, table tennis (TT) or squash stand out, and others of more recent creation and in the process of expansion and development such as paddle tennis, pickleball or beach tennis, have an excellent acceptance in today’s society. The characteristics of RS are of great social interest since they allow any type of population to practice them regardless of their culture, age, physical condition, gender, socio-economic level and physical or intellectual disability (Pradas and Herrero, 2015).

TT is one of the different disciplines that comes from real tennis, a sport that came from the *Jeu de Paume*, a twelfth century’s game played in England and the common ancestor of most of the RS which are currently practiced worldwide (Herrero et al., 2015). TT has evolved into a highly popular sport, recognized as one of the most well-known and widely played RS in the world. Currently, more than 300 million people practice TT, with at least 40 million licensed players (Pradas et al., 2021). Since its consolidation as a competitive sport in the first half of the twentieth century and its inclusion in the 1988’s Olympic Games, TT has been valued not only for its recreational nature and accessibility but also for its important physical and mental development benefits (International Table Tennis Federation (ITTF), 2022; Fuchs et al., 2018).

In recent years, TT has shown a positive impact in multiple areas. At health level, it has been demonstrated that its practice can improve coordination, agility and cardiovascular function, as well as promote mental sharpness, focus and concentration (Pradas et al., 2021; Liu et al., 2021). These characteristics have made TT a valuable tool for implementation in rehabilitation and disease prevention programs across a wide range of ages (Zhang et al., 2023; Smith and Lee, 2021).

In the educational context, TT has become an accepted and integral part of physical education (PE) programs due to its versatility and adaptability. It offers numerous benefits for students’ psychomotor and cognitive development. Various studies, including those by Han and Wang (2023), indicate that playing TT enhances both physical and mental health in adolescents. Its incorporation into education not only fosters the development of physical skills and social interaction but also promotes important values such as discipline, respect and teamwork. (Hernández and Robles, 2023). For these reasons, the need to implement TT has been highlighted in various locations (Herrero et al., 2016).

The use of TT in school curricula, both in primary and secondary education, has been minimally researched. It remains largely unclear whether this sport is effectively integrated into PE program. However, incorporating TT could provide significant benefits to the teaching and learning process in PE across various educational stages, broadening the opportunities for motor skills development, education, health and recreation. To support its educational settings’ integration, it is essential to have assessment tools to measure not only sports skills but also the cognitive and emotional benefits for students. Unfortunately, there is currently a lack of specific questionnaires designed to systematically and reliably evaluate these aspects (González-Devesa et al., 2024).

In this sense, this article aims to validate a questionnaire designed to measure the impact of TT in the educational context. The goal is to provide a reliable tool for teachers and professionals to analyze its implementation and evaluation in school programs related to PE.

## 2 Materials and methods

### 2.1 Methods

For sample selection, the main researcher of the study contacted a significant number of Spanish public high schools and then prepared a definitive list of participating educative centres. In the same way, the researcher explained to each secondary school’s potential participants the objectives of the study, its needs, the confidentiality and treatment of the data, and the procedures and implications of their participation. Subsequently, an informed consent for participation was provided which the interested parties had to read, fill in and sign if they agreeded to participate in the days following the explanatory session. Ethical standards, confidentiality, and data protection protocols were upheld at all times. The study responded to a quasi-experimental, ex post facto approach with incidence sampling using qualitative and quantitative analyses (Alto and Vallejo, 2015).

### 2.2 Participants

A total of 104 educational centres of Compulsory Secondary Education and Baccalaureate in the Region of Murcia agreed to participate. The target population was made up of a total of 384 PE teachers, achieving a total participation of 196, representing 50.5% of the total population belonging to this subject. According to gender, sample’s distribution was 142 males (72.4%) and 54 females (27.6%) with an average age between 31–35 years and 5–10 years of teaching experience. The whole study’s protocols and ethical considerations followed the Declaration of Helsinki, complying with all the criteria formulated (voluntary participation; informed consent and right to information; protection of personal data and full confidentiality; non-discrimination; free of charge; and the possibility of leaving the study at any time). The Ethics Committee of the Government of Aragon (ID: 12/2021) reviewed and approved the study.

### 2.3 Instruments

Within the framework of the survey research, a simple self-administered questionnaire was designed, in which the respondents themselves answered in writing without direct intervention from any person. The questionnaire is prepared to include questions on subject’s identification (age, sex, etc.) and content questions (Bisquerra, 1996). Moreover, different steps and actions are also considered in its construction to guarantee its reliability and validity. In this sense, Lozano and Fuentes, 2009 raised the importance of developing a questionnaire that accurately measures the study variable in a research process.

In this case, the questionnaire was developed using the Delphi method, a process that involves consulting experts to gather their opinions on a topic of interest during questionnaire design. The reasons to consider this method over the traditional one were: (a) It includes several rounds of questionnaires, interactions and queries related to and influenced by the previous round. (b) The answers are anonymous, which prevents the most prestigious participants from influencing the opinions of others. (c) In the Delphi method, no expert knows the identity of the others who are part of the group. (d) Each member of the group is equally considered. (e) It is based on the participant’s continuous feedback. Results are derived from a compendium of assessments and decisions on previous reviews. (f) The individual character of the participants is guaranteed.

The procedure followed to apply the Delphi method consisted of firstly designating a coordinating group, in this case, a single person. The coordinator, together with the principal investigator, stablished participants’ selection criteria, scheduled a calendar of actions, chose the e-mail as the technical mean to share the questionnaire, prepared the questionnaire, encouraged the participation of the experts, analyzed the responses, modified the questionnaire and prepared the following ones, provided appropriate feedback, interpreted the results, and finally, supervised the correct progress of the investigation.

A group of experts was then selected to participate in developing and validating the questionnaire. The group was made up of 16 people; active teachers belonging to different Spanish universities (*n* = 4), teachers from secondary schools (*n* = 8) and TT coaches with extensive experience at national and international level (*n* = 4). The experts’ selection was based on: (a) their professional profile, (b) research carried out, (c) publications on the subject, (d) proven experience, and finally, (e) their direct link with RSs, either in the educational field, the competitive field or in both situations, since they are considered to have relevant knowledge about the research topic.

From this moment on, a first questionnaire called the Racquet Sports Attitude Scale (RSAS) was prepared (Supplementary File 1), where a total of 62 potential questions were formulated, including demographic data, distributed in five categories: (a) difficulties in applying PE content, (b) positive attitude toward RSs, (c) benefits of TT, (d) facilitators for the application of TT; In the phase of preparing the initial questionnaire, the way of asking is specified, deciding on the number of questions, the order and their arrangement. After this preliminary design, a quantitative pilot study of reliability and validity with a sample of 20 professionals from different autonomous communities of Spain (Andalusia, Aragon, Castilla y León, Castilla laMancha, Catalonia, Valencian Community andMadrid) was carried out. This initial sample enabled the first checks on the reliability indices of the items, dimensions, and consistency of the questionnaire, resulting in Alpha indices ranging from 0.66 to 0.88. All this allowed three studies to be carried out (the first two converging into one) on a final sample of 196 PE professionals.

### 2.4 Statistical analysis

The first study (1a) was descriptive, comparative, and correlational in nature, aiming to establish the sociodemographic characteristics of the data. Reliability and validity were calculated to determine the psychometric properties of the preliminary questionnaire using Cronbach’s alpha as a measure of consistency. However, in subsequent analyses, this consistency was corroborated through ordinal Alpha data and parallel analyses.

The second study (1b) was based on an Exploratory Factor Analysis (EFA) that confirmed the number of dimensions of the questionnaire, as well as its reliability and validity. To achieve this, the Kaiser-Meyer-Olkin (KMO) index was calculated, and Bartlett’s test of sphericity was performed using the principal components method and oblimin rotation. Oblimin was selected considering the oblique relationship between the dimensions, rather than a closer relationship where a Varimax rotation would have been used.

Finally, the third study consisted of a Confirmatory Factor Analysis (CFA), which established the final number of items and their structure. Indicators of goodness-of-fit were examined, including Chi-square, the Comparative Fit Index (CFI), the Tucker-Lewis Index (TLI), and the Root Mean Square Error of Approximation (RMSEA) with a 90% confidence interval. Typical criteria for validity were values of CFI and TLI > 0.90, indicating good fit. RMSEA values of less than 0.08 and 0.06 were considered acceptable and excellent fits, respectively.

## 3 Results

The results are divided into two general studies. However, as previously indicated, the first study integrates descriptive and correlational studies with the first exploratory factor analysis (EFA). In this way, Study 1 reports on construct validity, internal consistency analysis, convergent validity, and exploratory factor analysis (EFA). As for the results of Study 2, they correspond to confirmatory factor analysis (CFA).

### 3.1 Study 1

#### 3.1.1 Construct validity

The primary objective of this study was the validation of the RSAS questionnaire. After the general design of the questionnaire based on the Delphi method with expert consultation, it was critically reviewed by a pilot sample of 20 PE teachers, followed by evaluation once again by the expert panel. This iterative quantitative and qualitative feedback process reduced the initial 62 questions to 42, including demographic data, and finally to 28 items without demographic data, distributed across five scales.

A principal components analysis with Oblimin rotation was chosen as the appropriate method after assuming oblique relationships between the dimensions of the questionnaire. The Kaiser-Meyer-Olkin (KMO) test yielded a value of 0.701, suggesting a moderate relationship between variables and indicating that the correlations among pairs of variables could be explained by the remaining variables. This necessitated a factor analysis, resulting in the distribution of five factors explaining 46.72% of the variance (F1 = 13.93%; F2 = 10.97%; F3 = 8.57%; F4 = 6.85%; F5 = 6.38%). Finally, the component matrix showed minimum values of 0.377 for item 19 in factor 4 and maximum values of 0.679 for item 18 in factor 3.

#### 3.1.2 Convergent validity

In turn, the different components of the dimensions were analyzed to check if there were significant correlations between them: (a) Benefits of table tennis; (b) Facilitators for the application of table tennis; (c) Barriers to the application of table tennis; (d) Difficulties in applying PE content and (e) Positive attitude toward racket sports. Table 1 shows the correlational analysis.

**Table 1:** Correlations among dimensions of the RSAS questionnaire.

	1	2	3	4
1. Benefits of TT				
2. Facilitators for the application of TT	0.48**			
3. Barriers to the application of TT	−0.16*	-		
4. Difficulties in applying PE content	0.10	−0.27	0.11	
5. Positive attitudes toward racquet sports	0.49**	0.32**	−0.28**	−0.04

TT, table tennis; PE, physical education.

The different components of the dimensions were analyzed to determine whether significant correlations existed among them: (a) Benefits of TT, (b) Facilitators for the application of TT, (c) Barriers to the application of TT, (d) Difficulties in applying PE content, and (e) Positive attitudes toward racquet sports. Table 1 presents the correlation analysis.

#### 3.1.3 Reliability

To corroborate the findings from the EFA and test the reliability of the instrument, ordinal alpha values were calculated for the set of items. Additionally, parallel analysis and polychoric matrices were conducted to confirm the five-factor structure. Global ordinal alpha values ranged from 0.65 to 0.72, with an average value of 0.70. Regarding the parallel analysis, the five-dimensional factor structure reported by the EFA model was confirmed. Ordinal alpha values between 0.70 and 0.90 indicate consistency in item measurement and, therefore, are suitable for measuring the same construct.

### 3.2 Study 2

**Figure 1:**
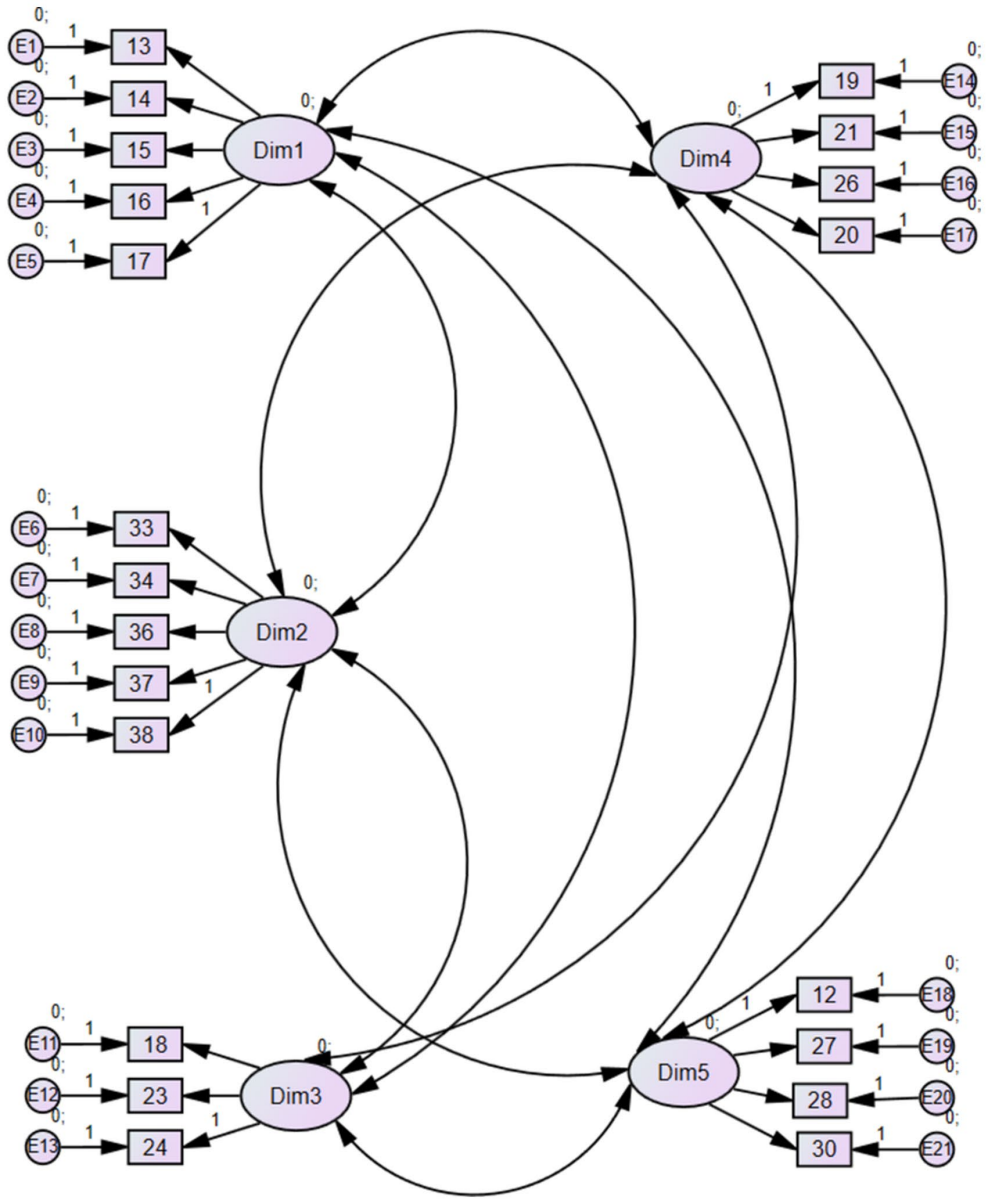
Five-factor model diagram.

The second study consisted of a confirmatory analysis to examine the internal structure of the scale. According to Batista and Coenders (2000), this was deemed the appropriate procedure to establish both general and item-specific validity and reliability. Two models were tested: the first, the original five-factor model (Figure 1), and a second, unidimensional model.

The purpose of this procedure was to compare the original model with alternative variants. To achieve this, a structural equation model was conducted using the maximum likelihood method. This yielded values for Chi-square, CFI, TLI, and AIC, which demonstrated that the original five-factor model with 21 final items outperformed the unidimensional model. The results showed values >0.90 for CFI and TLI, as well as RMSEA parameters below 0.06.

Therefore, considering the goodness-of-fit indices, the various parameters reported values indicating the sustainability of the initial five-factor model. Values obtained were X^2^ = 197.383; *p* < 0.001; CFI = 0.934; TLI = 0.923; and RMSEA = 0.030, which are significantly better than those yielded by the single-factor model (Table 2). Hence, it can be concluded that this structure was fully confirmed through statistical analyses derived from the different structural equation models and confirmatory factor analysis (CFA). All this suggests that the validated model optimally addresses the data provided and contributes to the validation of the questionnaire under study.

**Table 2:** Equation models and validity comparison.

	CFI	TLI	AIC	Chi-square	RMSEA
5 F Model	0.934	0.923	343.383	197.383	0.030
1 F Model	0.462	0.342	568.658	442.658	0.083

5 F, five-factor; 1 F, one-factor.

## 4 Discussion

The aim of this research is to validate a questionnaire designed to measure the impact of TT in the educational context in order to provide a reliable tool for teachers and professionals to analyze its implementation and evaluation in school programs related to PE.

In this regard, this article explores and validates the RSAS questionnaire, contributing to the growing body of research dedicated to developing and adapting specific instruments to measure various variables in educational and sports contexts. Considering the target population of study and analysis, studies such as the one conducted by Gudiel-Hermoza et al. (2021), in which the ASQ-3 questionnaire was adapted for the early detection of developmental problems in children, concluded the need to design tools that address the cultural and linguistic particularities of target populations.

On the other hand, works such as those developed by Rodríguez-Rivadulla et al. (2019), focused on emerging sports, or those by Pi et al. (2022), centered on sports well-being, clearly highlight the usefulness of adapted questionnaires to address gaps in data systematization or to promote safer and healthier practices—areas of great interest in the educational field today. Similarly, other questionnaires such as the NSKQ-BR, which evaluates knowledge of nutrition in sports (Sousa et al., 2024), or the APSQ-Cro, which measures various psychological aspects in athletes (Sore et al., 2024), expand the scope of analysis expectations in sports by developing evaluation tools for even more specific areas. In line with this, studies by Trpkovici et al. (2023) developed and validated a questionnaire to measure athlete anxiety in a multidimensional way, addressing other aspects of interest such as concentration and self-confidence, or the work by Delrue et al. (2019), which refined a questionnaire designed to assess different training styles through a dynamic and situational approach.

However, as can be seen, and to the best of our knowledge of the scientific literature published, there are few questionnaires that assess specific sports applied to the school environment and their impact. In this research line, a pioneering questionnaire can be highlighted, attempting to analyze the situation of racquet sports in PE at the secondary education level in the Murcia region, particularly focusing on TT as a school subject (Herrero, 2015). Considering the importance of further exploring the educational field and the sports content used, this study differs from other publications where questionnaires have been developed and validated, as it addresses the integration of racquet sports in a very specific context—educational settings—focusing on TT and analyzing its significance in the teaching and learning process as educational content. This is an area that has been scarcely explored and is largely unknown but holds great potential and motivation to enrich the PE curriculum.

Given the background outlined, the need of creating a new tool through the development and validation of the RSAS questionnaire arises. To meet this demand in the educational and sports contexts, a systemic and methodological validation system was carried out, which included expert design, qualitative assessment, pilot testing, and both quantitative and qualitative analysis, as well as exploratory analysis of the proposed model and confirmatory analysis of the proposed five-factor model and an alternative unidimensional model. The results obtained in this process confirmed the five-factor structure in both the exploratory analysis and the parallel analysis, indicating good factor loadings and statistically valid alpha values for each item and its grouping into dimensions (Michel et al., 2013).

Furthermore, the validation results indicated that the factor structure of the RSAS scale was able to explain 46.72% of the variance, which, along with the previously described values, supports the consistency of the scale, indicating its validity and reliability. This finding reinforces what was reported by Sousa et al. (2024) and Sore et al. (2024) about the importance of establishing robust models to evaluate complex constructs in various fields, such as nutritional knowledge and athletes’ mental health.

Additionally, the relationship between factors was examined through correlation data, and although no correlation was found between dimensions for all pairings, this was supplemented with other control elements for oblique relationships, such as the Oblimin rotation method, parallel analysis, polychoric matrices, and evaluation of ordinal alpha parameters above Cronbach’s Alpha. This led to the conclusion of the effectiveness of each item and dimension in measuring the same global construct.

Supporting the validation results obtained in this questionnaire are several works, such as those by Trpkovici et al. (2023) and Delrue et al. (2019), which emphasize the importance of using exploratory and confirmatory factor analyses to ensure the psychometric quality of questionnaires, as was done in this study. Taken together, these studies illustrate the positive impact of validated tools in educational and sports practice, promoting evidence-based strategies that optimize the physical, emotional, and cognitive development of participants.

## 5 Limitations

Although the validation analyses have reached optimal conclusions regarding the tool’s robustness and validation, certain limitations should not be overlooked, such as the need to expand the participant sample, as well as its educational and geographical diversity. This would allow for conclusions not only about the validity of the instrument, which has already been confirmed by this study, but also regarding the actual use of the tool and its results. All of this would lead to a greater understanding of the current status of racquet sports in PE across different educational stages: Primary Education, Secondary Education, High School, and University.

## 6 Conclusion

The RSAS questionnaire is designed to assess teachers’ opinions on the practice and impact of racquet sports, particularly TT, as an educational content in PE classes.

The results obtained from the design process of the RSAS questionnaire confirm that the five-factor structure, both in exploratory analysis and parallel analysis, presents adequate saturation values and statistically valid alpha values for each item and its grouping into dimensions. On the other hand, the validation results indicate that the factorial structure of the RSAS scale was able to explain 46.72% of the variance, which, combined with the previously described values, supports the consistency of the scale, indicating its validity and reliability.

Moreover, the relationship between factors was also examined through correlation data. Although no correlation was found between dimensions for all pairings, this was supplemented with the use of other control elements for oblique relationships, such as the Oblimin rotation method, parallel analysis, polychoric matrices, and evaluation of ordinal alpha parameters above Cronbach’s alpha. This led to the conclusion of the effectiveness of each item and dimension in measuring the same global construct.

This study improves the evaluation of racquet sports in the educational field through the RSAS questionnaire, especially the use and impact of TT, allowing for an understanding of teachers’ opinions about this sport in terms of its implementation or lack thereof in PE classes, addressing attitudes, benefits, barriers, and difficulties in using this sports content. Future research is expected to use the RSAS to assess the opinions of teachers from different educational contexts to understand the level of use of these sports and take appropriate measures for their correct and proper use at any level in PE classes.

## Data Availability

The original contributions presented in the study are included in the article/supplementary material, further inquiries can be directed to the corresponding aurhor.
