# Correction: Development and validation of a questionnaire for table tennis teaching in physical education

**DOI:** 10.3389/fpsyg.2025.1675905

**Published:** 2025-09-19

**Authors:** Miguel Ángel Ortega-Zayas, Antonio José Cardona-Linares, Alberto Quílez, Alejandro García-Giménez, Miguel Lecina, Francisco Pradas

**Affiliations:** ^1^ENFYRED Research Group, Faculty of Health and Sports Sciences, University of Zaragoza, Huesca, Spain; ^2^Faculty of Human Sciences and Education, University of Zaragoza, Zaragoza, Spain; ^3^Faculty of Health Sciences, ENFYRED Research Group, University of Zaragoza, Zaragoza, Spain

**Keywords:** racket sports, questionnaire, physical education, teacher skills, educational content

Supplemental material “Supplementary File 1 - RSAS_Questionnaire_2025.docx” was omitted. The file has now been published.

A correction has been made to the section **2 Materials and methods**, *2.3 Instruments*, paragraph 5 to include the citation to the supplementary material.

“From this moment on, a first questionnaire called the Racquet Sports Attitude Scale (RSAS) was prepared (Supplementary File 1), where a total of 62 potential questions were formulated, including demographic data, distributed in five categories: (a) difficulties in applying PE content, (b) positive attitude toward RSs, (c) benefits of TT, (d) facilitators for the application of TT; In the phase of preparing the initial questionnaire, the way of asking is specified, deciding on the number of questions, the order and their arrangement. After this preliminary design, a quantitative pilot study of reliability and validity with a sample of 20 professionals from different autonomous communities of Spain (Andalusia, Aragon, Castilla y León, Castilla la Mancha, Catalonia, Valencian Community and Madrid) was carried out. This initial sample enabled the first checks on the reliability indices of the items, dimensions, and consistency of the questionnaire, resulting in Alpha indices ranging from 0.66 to 0.88. All this allowed three studies to be carried out (the first two converging into one) on a final sample of 196 PE professionals.”

The original version of this article has been updated.

