# Supplementary material for: Development and validation of a questionnaire for table tennis teaching in physical education
Source: Front Psychol. 2025 Jan 24;16:1550061. doi: 10.3389/fpsyg.2025.1550061 (PMC11824272; doi:10.3389/fpsyg.2025.1550061)
Supplement: Supplementary file 1 [file Supplementary_file_1.docx]

**RACKET SPORTS ATTITUDE SCALE (RSAS)**

***SECTION 1*. GENERAL QUESTIONS**

1. Sex

|  | Male |  | Female |
| --- | --- | --- | --- |

2. Age

| **_______** | years |
| --- | --- |

3. Academic qualification

|  | Bachelor's Degree in Education with Physical Education specialization |
| --- | --- |
|  | Bachelor's Degree in Physical Activity and Sports Sciences |
|  | Bachelor's Degree in Education with another specialization |
|  | Other Bachelor's Degree |
|  | Other: ____________________________________________ |

4. Administrative status

|  | Permanent assignment |  | Interim |
| --- | --- | --- | --- |
|  | Awaiting assignment |  | Other: ___________________ |
|  | Internship |  |  |

5. Position at the school

|  | Teacher |  | Head of Department |
| --- | --- | --- | --- |
|  | Principal |  | Homeroom Teacher |
|  | Head of Studies |  | Other |
|  | Secretary |  |  |

6. Teaching experience

| **_______** | years |
| --- | --- |

7. Type of school where you work at

|  | Public |  | Semi-private |  | Private |
| --- | --- | --- | --- | --- | --- |

8. Name of the town/city and province where the school is located

| ______________________________________________ |  |
| --- | --- |

***SECTION 2*. SPECIFIC QUESTIONS**

9. The school has appropriate facilities to set up game table tennis and either leave them set up or store them after use

|  | Strongly disagree |  | Agree |
| --- | --- | --- | --- |
|  | Disagree |  | Strongly agree |

10. Indicate the number of game table tennis available at your school

|  | 0 tables |  | 3–4 tables |
| --- | --- | --- | --- |
|  | 1–2 tables |  | More than 4 tables |

11. If you have game tables tennis, indicate the frequency of their use

|  | We don’t have tables |  | Moderate use |
| --- | --- | --- | --- |
|  | No use |  | Frequent use |
|  | Little use |  |  |

12. I have taken training courses or participated in activities related to the following racquet sports

|  | Tennis |
| --- | --- |
|  | Pelota (*frontón, trinquete* or *frontenis*) |
|  | Padel |
|  | Table tennis |
|  | Squash |
|  | Badminton |
|  | Alternative sports (shuteball, basic setback games, others) |
|  | None |

13. The didactic materials (reference books, videos, etc.) available for my classes are related to the following sports

|  | Tennis |
| --- | --- |
|  | Pelota (*frontón, trinquete* or *frontenis*) |
|  | Padel |
|  | Table tennis |
|  | Squash |
|  | Badminton |
|  | Alternative sports (shuteball, basic setback games, others) |
|  | None |

***SECTION 3*. DIFFICULTIES IN IMPLEMENTING PHYSICAL EDUCATION CONTENT**

Regarding Physical Education (PE) content

|  | Strongly disagree | Disagree | Agree | Strongly agree |
| --- | --- | --- | --- | --- |
| 14. I am reluctant to introduce certain curricular contents in PE because I consider them dangerous |  |  |  |  |
| 15. I am reluctant to introduce content in PE that is not well known |  |  |  |  |
| 16. I am reluctant to introduce new content when my knowledge to teach it is limited |  |  |  |  |
| 17. I am reluctant to introduce PE content that involves too much work |  |  |  |  |
| 18. I am reluctant to introduce some PE content because I don't have enough time |  |  |  |  |
| 19. I am reluctant to introduce PE content that is economically costly |  |  |  |  |

***SECTION 4*. POSITIVE ATTITUDES TOWARDS RACQUET SPORTS**

Regarding Physical Education content

|  | Strongly disagree | Disagree | Agree | Strongly agree |
| --- | --- | --- | --- | --- |
| 20. Racket sports are a highly recommended practice for students |  |  |  |  |
| 21. Racket sports included in teaching programs contribute to students' physical, mental, and social well-being |  |  |  |  |
| 22. The popularity of racket would justify giving them greater emphasis at school |  |  |  |  |
| 23. I would support legislation that explicitly includes "racket sports" (including table tennis) as educational content |  |  |  |  |

24. Do you include any unit related to a racket sports (other than table tennis) in your PE curriculum?

|  | Yes | No |
| --- | --- | --- |
| Tennis |  |  |
| Squash |  |  |
| Pelota (*frontón*, *trinquete* or *frontenis*) |  |  |
| Badminton |  |  |
| Padel |  |  |
| Alternative sports (shuteball, basic setback games, others) |  |  |

***SECTION 5*. BENEFITS OF TABLE TENNIS**

Indicate your agreement with the following statements regardless of whether table tennis is included in your classes

|  | Strongly disagree | Disagree | Agree | Strongly agree |
| --- | --- | --- | --- | --- |
| 25. Playing table tennis involves less risk of injury compared to other racket sports |  |  |  |  |
| 26. Table tennis is a suitable sport for students with physical problems |  |  |  |  |
| 27. Table tennis is a recommended sport for students with special educational needs |  |  |  |  |
| 28. Table tennis is a very safe sport (no physical risks) for primary school students |  |  |  |  |
| 29. In PE classes, using table tennis increases students’ interest and engagement |  |  |  |  |
| 30. Table tennis is a highly recommended activity for PE in primary education |  |  |  |  |

31. Do you include any unit related to table tennis in your PE Department's curriculum?

|  | Yes (continue to question 32) |
| --- | --- |
|  | No (skip to question 39) |

***SECTION 6*. FACILITATORS FOR THE IMPLEMENTATION OF TABLE TENNIS**

Indicate your agreement with the following statements

|  | Strongly disagree | Disagree | Agree | Strongly agree |
| --- | --- | --- | --- | --- |
| 32. I chose to include table tennis in PE due to its value for learning |  |  |  |  |
| 33. I chose to include table tennis in my classes because of material availability |  |  |  |  |
| 34. I included table tennis because of student demand |  |  |  |  |
| 35. I included table tennis because I have appropriate facilities |  |  |  |  |
| 36. I included table tennis because it is a well-known or commonly played sport |  |  |  |  |
| 37. I included table tennis because it is easy to implement in PE classes |  |  |  |  |

38. Is there any other reason why you chose to incorporate table tennis in Physical Education?

| ________________________________________________________________ |
| --- |
| ________________________________________________________________ |
| ________________________________________________________________ |
| ________________________________________________________________ |
| ________________________________________________________________ |

***SECTION 7*. BARRIERS TO THE IMPLEMENTATION OF TABLE TENNIS**

Indicate your agreement with the following statements:

|  | Strongly disagree | Disagree | Agree | Strongly agree |
| --- | --- | --- | --- | --- |
| 39. I do not include table tennis activities because they do not provide valuable learning experiences. |  |  |  |  |
| 40. I do not include table tennis due to safety concerns. |  |  |  |  |
| 41. I do not include table tennis because it is not motivating. |  |  |  |  |
| 42. I do not include table tennis due to wear and tear on the materials. |  |  |  |  |
| 43. I do not include table tennis because students do not request it. |  |  |  |  |
| 44. I do not include table tennis because it is not a well-known sport. |  |  |  |  |

45. Is there any other reason why you chose not to include table tennis in Physical Education?

| ________________________________________________________________ |
| --- |
| ________________________________________________________________ |
| ________________________________________________________________ |
| ________________________________________________________________ |
| ________________________________________________________________ |

**THANK YOU FOR YOUR COLLABORATION**
